# Supplementary material for: Strong Plasmon–Exciton Coupling in Ag Nanoparticle—Conjugated Polymer Core-Shell Hybrid Nanostructures
Source: Polymers (Basel). 2020 Sep 19;12(9):2141. doi: 10.3390/polym12092141 (PMC7570213; doi:10.3390/polym12092141)
Supplement: Supplementary file 1 [file polymers-12-02141-s001.pdf]

Supporting Information

Strong Plasmon-Exciton Coupling in Ag Nanoparticle – Conjugated Polymer Core-Shell Hybrid Nanostructures

Christopher E. Petoukhoff,[1,2*] *Keshav M. Dani,*[2] *Deirdre M. O'Carroll,*[1,3*]

1. Department of Materials Science and Engineering, Rutgers University, 607 Taylor Rd., Piscataway, NJ 08854, USA.

2. Femtosecond Spectroscopy Unit, Okinawa Institute of Science and Technology Graduate University, Onna, Okinawa 904-0495, Japan

3. Department of Chemistry and Chemical Biology, Rutgers University, 610 Taylor Rd., Piscataway, NJ 08854, USA.

Keywords: conjugated polymer, exciton, plasmon, vibrationally-dressed, strong coupling

Mie theory calculations – from Bohren and Huffman[1]

Analytical Mie theory was used to corroborate the numerical FDTD simulations. The equations employed were those used to calculate the extinction and scattering cross-sections from a sphere of radius r and from a coated sphere with linear, isotropic, homogeneous complex refractive indices for both the sphere (N_1) and coating (N_2) embedded in a dielectric medium with index, N. The scattering (C_{scat}) and extinction (C_{ext}) cross-sections are given by:

$$C_{\mathrm{scat}} = \frac{2\pi}{k^2}\sum_{n=1}^{\infty}(2n+1)\left(|a_n|^2 + |b_n|^2\right) \quad \text{S1}$$

$$C_{\mathrm{ext}} = \frac{2\pi}{k^2}\sum_{n=1}^{\infty}(2n+1)\,\mathrm{Re}\{a_n + b_n\} \quad \text{S2}$$

where k is the wavevector $k = 2\pi N/\lambda$, λ is the wavelength, and a_n and b_n are the scattering coefficients obtained from expansion of the spherical harmonic solutions to the vector wave equations. Assuming the permeability of the particle and the surrounding medium are the same, the scattering coefficients of a bare sphere are given by:

$$a_n = \frac{m\psi_n(mx)\psi_n'(x) - \psi_n(x)\psi_n'(mx)}{m\psi_n(mx)\xi_n'(x) - \xi_n(x)\psi_n'(mx)} \quad \text{S3}$$

$$b_n = \frac{\psi_n(mx)\psi_n'(x) - m\psi_n(x)\psi_n'(mx)}{\psi_n(mx)\xi_n'(x) - m\xi_n(x)\psi_n'(mx)} \quad \text{S4}$$

where m and x are the relative refractive index and size parameter, respectively, given by:

$$m = \frac{k_1}{k} = \frac{N_1}{N} \qquad x = kr = \frac{2\pi N r}{\lambda} \quad \text{S5}$$

and ψ_n and ξ_n are the Ricatti-Bessel functions, which help to simplify the calculations. The Ricatti-Bessel functions were calculated from the Bessel functions of the first and third kinds, *i.e.*, $J_n(\rho)$ and $H_n^{(1)}(\rho)$, respectively, using the relationships:

$$\psi_n(\rho) = \sqrt{\frac{\rho\pi}{2}} J_{n+\frac{1}{2}}(\rho) \qquad \text{S6}$$

$$\xi_n(\rho) = \sqrt{\frac{\rho\pi}{2}} H^{(1)}_{n+\frac{1}{2}}(\rho) \qquad \text{S7}$$

For a coated sphere, the scattering coefficients are slightly modified:

$$a_n = \frac{\psi_n(y)\left[\psi_n'(m_2 y) - A_n \chi_n'(m_2 y)\right] - m_2 \psi_n'(y)\left[\psi_n(m_2 y) - A_n \chi_n(m_2 y)\right]}{\xi_n(y)\left[\psi_n'(m_2 y) - A_n \chi_n'(m_2 y)\right] - m_2 \xi_n'(y)\left[\psi_n(m_2 y) - A_n \chi_n(m_2 y)\right]} \qquad \text{S8}$$

$$b_n = \frac{m_2 \psi_n(y)\left[\psi_n'(m_2 y) - B_n \chi_n'(m_2 y)\right] - \psi_n'(y)\left[\psi_n(m_2 y) - B_n \chi_n(m_2 y)\right]}{m_2 \xi_n(y)\left[\psi_n'(m_2 y) - B_n \chi_n'(m_2 y)\right] - \xi_n'(y)\left[\psi_n(m_2 y) - B_n \chi_n(m_2 y)\right]} \qquad \text{S9}$$

where m_1 and m_2 are the refractive indices of the core and coating relative to the surrounding medium, $y = k(r+h)$, h is the shell thickness, χ_n is the Ricatti-Bessel function of the second kind, and A_n and B_n are given by:

$$A_n = \frac{m_2 \psi_n(m_2 x)\psi_n'(m_1 x) - m_1 \psi_n'(m_2 x)\psi_n(m_1 x)}{m_2 \chi_n(m_2 x)\psi_n'(m_1 x) - m_1 \chi_n'(m_2 x)\psi_n(m_1 x)} \qquad \text{S10}$$

$$B_n = \frac{m_2 \psi_n(m_1 x)\psi_n'(m_2 x) - m_1 \psi_n(m_2 x)\psi_n'(m_1 x)}{m_2 \chi_n'(m_2 x)\psi_n(m_1 x) - m_1 \psi_n'(m_1 x)\chi_n(m_2 x)} \qquad \text{S11}$$

Note that χ_n is related to the Bessel function of the second kind, Y_n, similar to the previous Ricatti-Bessel functions:

$$\chi_n(\rho) = -\sqrt{\frac{\rho\pi}{2}} Y_{n+\frac{1}{2}}(\rho) \qquad \text{S12}$$

Equations S1-S12 were employed to calculate the scattering and extinction cross-sections analytically. The absorption cross-section was calculated by: $C_{\text{abs}} = C_{\text{ext}} - C_{\text{scat}}$. The cutoff value for the infinite series summation (*i.e.*, n_{stop}) was determined by the integer closest to:

$$n_{\text{stop}} = \max(x) + 4\max(x)^{\frac{1}{3}} + 2 \qquad \text{S13}$$

A wavelength step size of 1 nm was used for the analytical Mie theory calculations. We note that the electrostatic (*i.e.*, Rayleigh) approximation was not valid for the particle sizes used throughout the main text since $r \sim \lambda$ (see Figure S1).

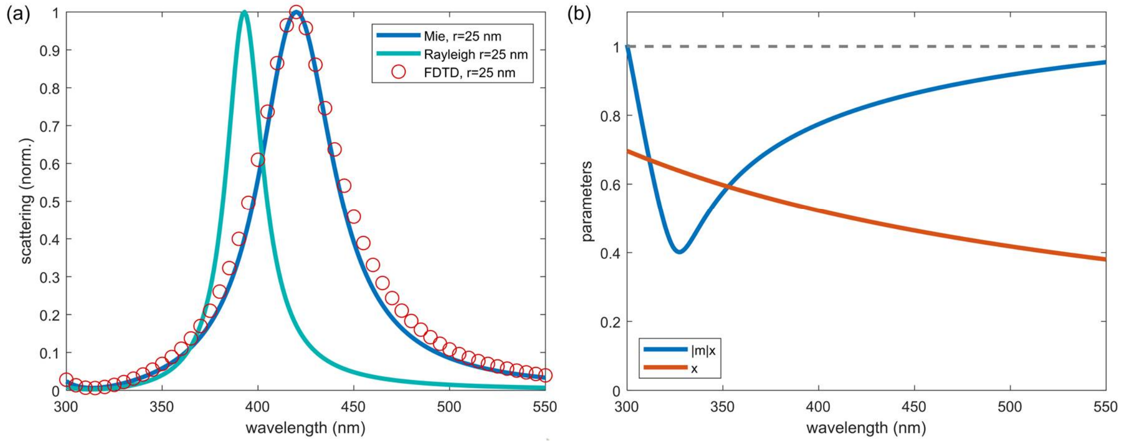

Figure S1. Validity of Mie vs Rayleigh scattering and FDTD simulations. (a) Calculated normalized scattering cross-section obtained for a Ag sphere with radius (r) of 25 nm in a medium of water (medium relative permittivity = 1.77). The complex relative permittivity for Ag was the same for all three types of calculations. Rayleigh and Mie calculations were conducted via the equations described by Bohren and Huffman,[1] which are shown in Equations S1-S12 for Mie theory. (b) Since x and $|m|x$ were both nearly 1, the electrostatic (*i.e.*, Rayleigh) approximation was not valid.

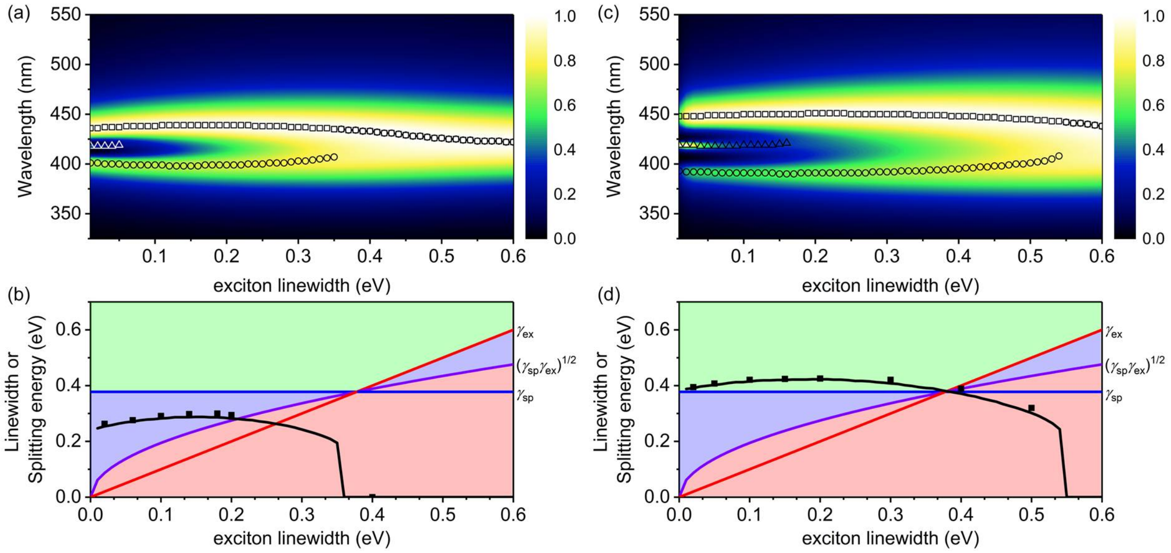

Figure S2. Variation of resonance linewidth, $\hbar\gamma$, for core-shell structures with a single Lorentzian oscillator shell with r = 25 nm, ε_∞ = 1.77, λ_0 = 420 nm, f = 0.05, and (a,b) h = 5 nm or (c,d) h = 40 nm. (a,c) Normalized scattering cross-section calculated using Mie theory. The colorbar represents the normalized scattering intensity, and the open symbols represent the peaks of the dominant modes. (b,d) Splitting energy for hybrid plasmon-exciton modes calculated using FDTD simulations (symbols) and Mie theory (black line). The background shading represents the coupling regimes (green - strong coupling; blue - intermediate coupling; red - weak coupling) as defined by the linewidths of the surface plasmon (γ_{sp} - blue line), the exciton (γ_{ex} - red line), and the relationship: $\sqrt{\gamma_{sp}\gamma_{ex}}$ (purple line), given in Equation 5.

We investigated the influence of the exciton linewidth ($\hbar\gamma$) on the scattering and coupling strength from core-shell NPs with r = 25 nm, ε_∞ = 1.77, λ_0 = 420 nm, and f = 0.05 for thin (5 nm) and thick (40 nm) shells (Figure S2). For narrow $\hbar\gamma$ values (< 0.1 eV), the scattering spectra displayed the third shell mode at the resonance wavelength (Figure S2a,c), particularly for 40-nm-thick shells. As $\hbar\gamma$ increased, the splitting between the long- and short-wavelength plasmon-exciton modes increased slightly for both thin and thick shells, but the shell mode peak decreased in intensity. For 5-nm-thick shells, the coupling transitioned from the intermediate to weak coupling regime as $\hbar\gamma$ increased beyond 0.2 eV (Figure S2b); for 40-nm-thick shells, the coupling was strong for all $\hbar\gamma \leq$ 0.38 eV. As $\hbar\gamma$ increased further, the coupling transitioned to the weak

coupling regime, the hybrid modes continued to broaden, until eventually they overlapped so strongly that they merged to one, uncoupled peak. This was due to the large $\hbar\gamma$ influencing the shell to become only weakly dispersive within the visible regime, and the shell acted as just an absorptive filter. Thus, within the range of $\hbar\gamma$ typical for single oscillators of conjugated polymers, the coupling was in the strong coupling regime under the conditions of a single oscillator shell with h = 40 nm and f = 0.05. We note that f = 0.05 is a relatively low value for single oscillators of conjugated polymers, and most polymers exhibit multi-oscillator vibronic structure in their optical properties.

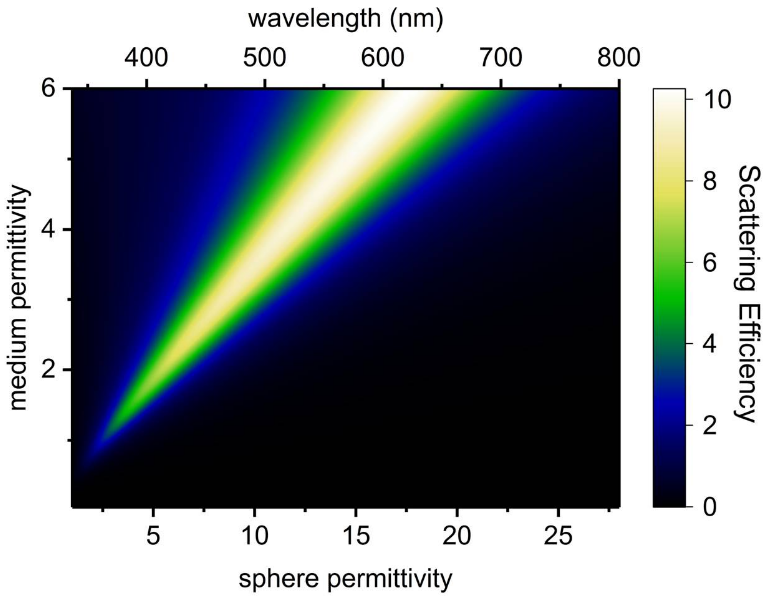

Figure S3. Scattering efficiency (*i.e.*, $Q_{\text{scat}} = C_{\text{scat}} / \left(\pi r^2\right)$) calculated using analytical Mie theory for a bare Ag sphere with radius, r = 25 nm embedded in a uniform dielectric medium with varying real relative permittivity. The magnitude of the complex relative permittivity of Ag is shown on the x-axis as a reference.

All of the coupled plasmon-exciton hybrid modes exhibited asymmetry in their intensity: the long wavelength mode was consistently more intense than the short wavelength mode,

particularly as the coupling strength increased. The reason for this asymmetry arose from the asymmetry in the real part of the relative permittivity of the shell (Figure 1d,e). For large oscillator strengths and multiple vibronic resonances, the real part of the relative permittivity can be as low as 2 for $\lambda < \lambda_0$, and as large as 5 for $\lambda > \lambda_0$. This asymmetry in the real part of the permittivity has two effects: 1) the larger permittivity for $\lambda > \lambda_0$ red-shifts the long wavelength peak further while the lower permittivity for $\lambda < \lambda_0$ blue-shifts the short wavelength peak; 2) higher permittivity values and longer wavelengths have higher scattering efficiencies than lower permittivities and shorter wavelengths (Figure S3). Thus, the asymmetry in the real part of the relative permittivity of the shell is responsible for the increased intensity of the long wavelength plasmon-exciton mode, in some cases (*e.g.*, very large oscillator strength or multiple vibronic resonances), resulting in the short wavelength plasmon-exciton mode being almost fully diminished.

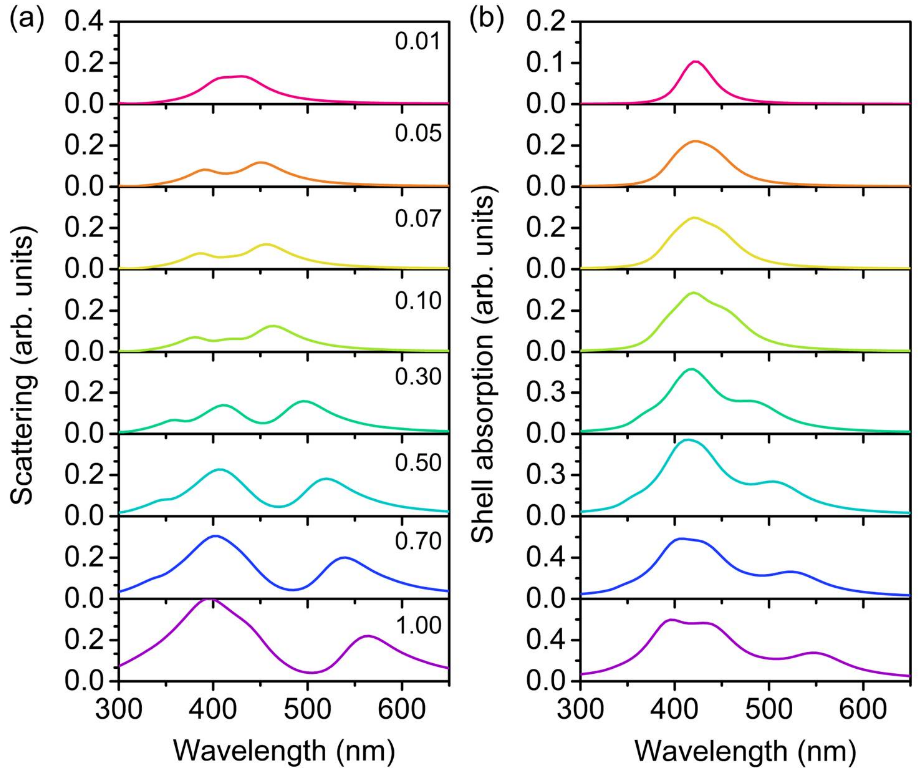

Figure S4. Variation of oscillator strength, *f*, for core-shell structures with a single Lorentzian oscillator shell with h = 40 nm, ε_∞ = 1.77, λ_0 = 420 nm, and $\hbar\gamma$ = 0.30 eV. (a) Non-normalized scattering intensity; and (b) shell absorption calculated using FDTD simulations.

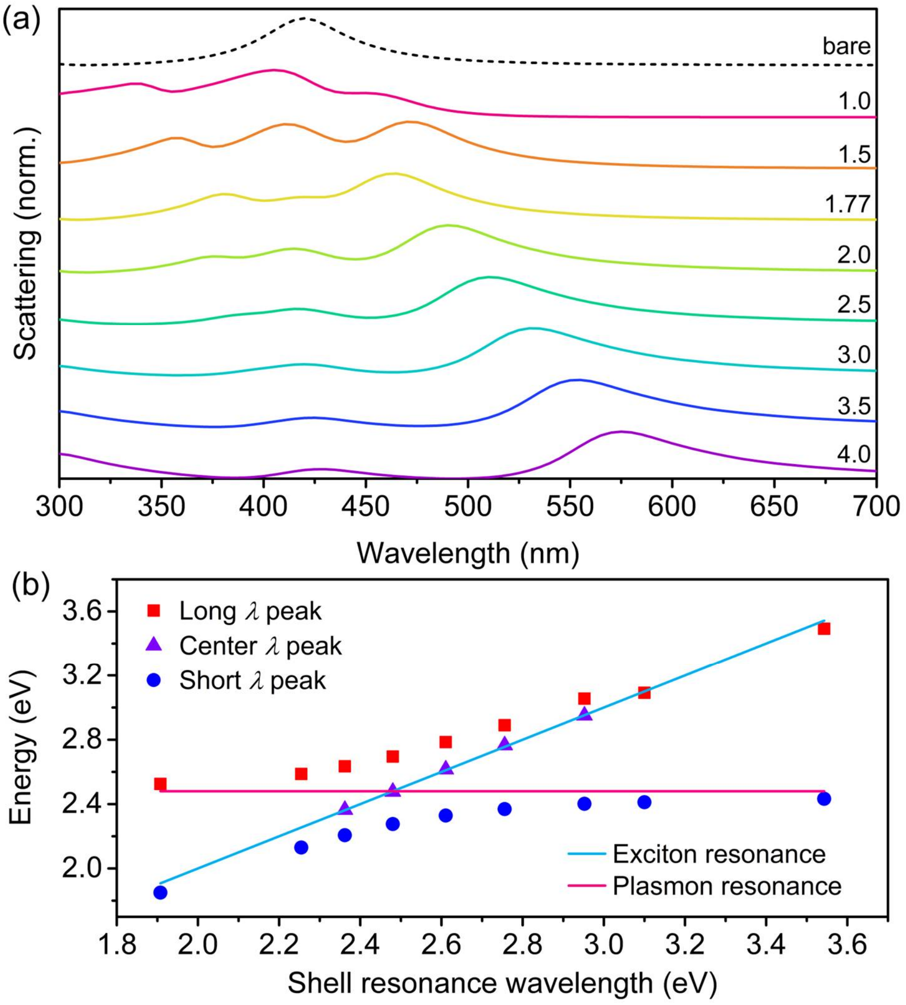

Figure S5. (a) Normalized scattering spectra for variation of high-frequency relative permittivity, ε_∞, for core-shell structures with a single Lorentzian oscillator shell with $r = 25$ nm, $h = 40$ nm, $\lambda_0 = 420$ nm, $f = 0.10$, and $\hbar\gamma = 0.30$ eV. (b) Dispersion curve showing the peak energies of the hybrid states from the core-shell structures for varying λ_0 of the shell for $r = 25$ nm, $h = 40$ nm, $f = 0.10$, $\hbar\gamma = 0.15$ eV, and $\varepsilon_\infty = 3.0$. The uncoupled exciton resonance energies of the shell and the fitted LSPR energy are overlaid as solid lines. Calculations here were conducted using FDTD simulations.

For a core-shell structure with a fixed λ_0, as ε_∞ increased, the long wavelength scattering peak increased in intensity and red-shifted; the short wavelength peak also red-shifted slightly to 430 nm for $\varepsilon_\infty = 4.0$ (Figure S5a). However, since there was a flat increase in the real part of the relative permittivity of the conjugated polymer shell, the uncoupled LSPR of the AgNP could no

longer be taken as the wavelength of the bare AgNP in a medium with a background relative permittivity (ε_{bkgd}) of 1.77. The wavelength of the uncoupled LSPR of the AgNP was determined by fixing r = 25 nm and varying λ_0 of the conjugated polymer shell for a fixed ε_∞ = 3.0 (Figure S5b). From this dispersion plot, the red-shifted uncoupled LSPR was determined to occur at 500 nm (*i.e.*, 2.48 eV), which was the same wavelength for the LSPR of a AgNP in a medium with ε_{bkgd} = 3.0 (Figure 5a). Thus, for the variation of ε_∞ in the main text, λ_0 for each ε_∞ was first determined by simulating bare AgNPs immersed in background permittivities equal to ε_∞, then λ_0 was determined from the LSPR wavelengths and was adjusted for the results shown in Figure 5.

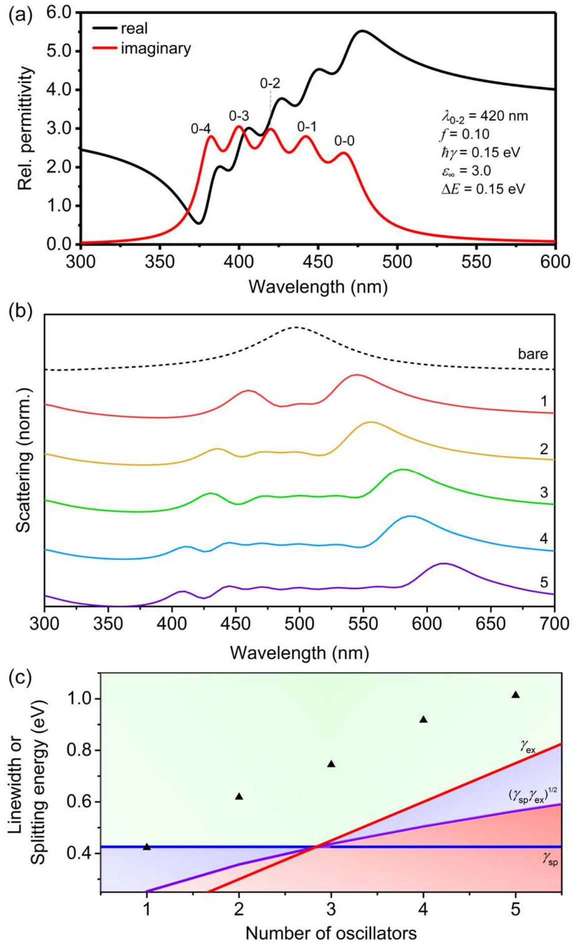

Figure S6. (a) Real and imaginary parts of the relative permittivity for a model conjugated polymer comprised of m = 5 oscillators. The complex relative permittivity was calculated using Equation 2 in the main text and the parameters included in (a). Symbols used: 0-v = absorption transition from $S_0 \rightarrow S_1$, into the vth vibrational level; $\lambda_{0\text{-}2}$ = resonance wavelength for 0-2 transition; f = oscillator strength; $\hbar\gamma$ = resonance linewidth; ε_∞ = high-frequency relative permittivity; ΔE = spacing between oscillators. (b) Normalized scattering spectra for variation of number of oscillators, m, for core-shell structures with shell relative permittivity described by the summation of Lorentzian model and with h = 40 nm, f = 0.10; $\hbar\gamma$ = 0.15 eV; ΔE = 0.15 eV; and ε_∞ = 3.0. The center oscillator (*i.e.*, 0-0, 0-0, 0-1, 0-1, and 0-2 transitions for 1-5 oscillators, respectively) was resonant with the uncoupled LSPR of the AgNP (*i.e.*, 500 nm), with additional vibrational modes either blue- or red-shifted from the uncoupled LSPR. (c) Splitting energy for hybrid exciton-plasmon modes for variation of the number of oscillators. The background shading represents the coupling regimes (green - strong coupling; blue - intermediate coupling; red - weak coupling) as defined by the linewidths of the surface plasmon (γ_{sp} - blue line), the exciton (γ_{ex} - red line), and the relationship: $\sqrt{\gamma_{sp}\gamma_{ex}}$ (purple line), given in Equation 5. The peak splitting was obtained from the scattering spectra as the difference in energy between the highest and lowest energy hybrid modes. All calculations were conducted using FDTD simulations.

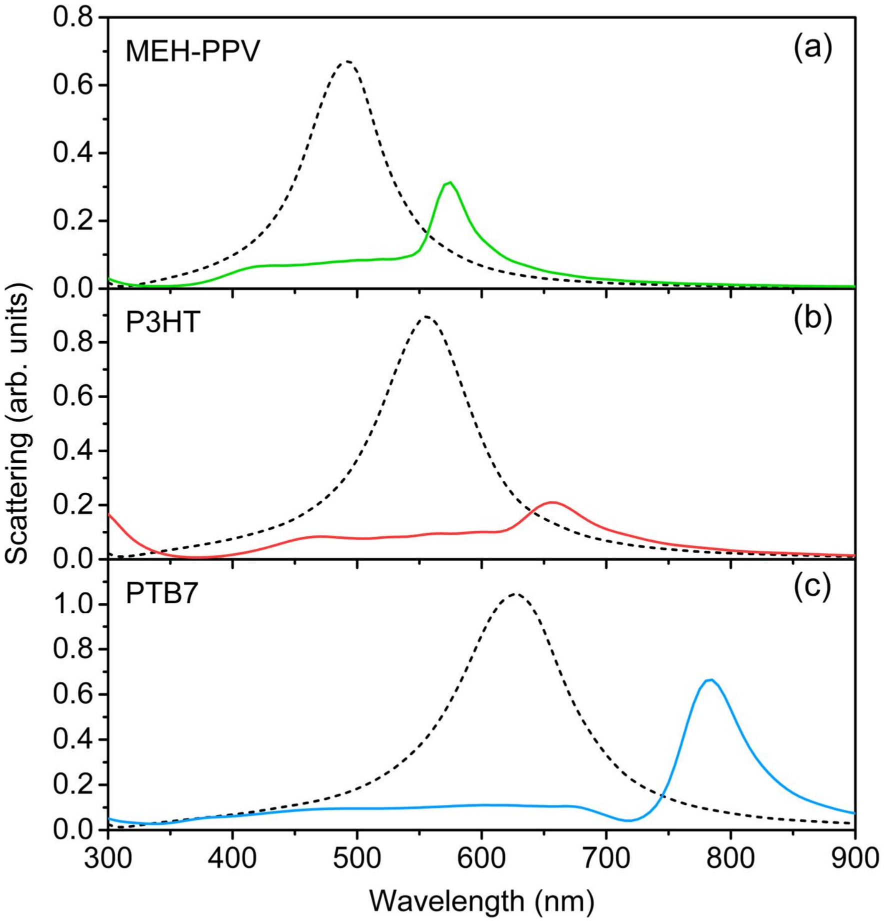

Figure S7. Non-normalized scattering spectra for prolate Ag nanorod core-shell structures with dispersive relative permittivities for common conjugated polymer materials used as the shells for $h = 40$ nm. The nanorod short axes were 25 nm and the long axes were: 28 nm (a; MEH-PPV); 33 nm (b; P3HT); and 37 nm (c; PTB7). Same data as shown in Figure 7a-c of the main text, except not normalized. Calculations conducted using FDTD simulations.

References

1. Bohren, C. F.; Huffman, D. R., *Absorption and Scattering of Light by Small Particles*. WILEY-VCH Verlag GmbH & Co. KGaA: Weinheim, Germany, 2004.
